# Combatting SARS-CoV-2 With Digital Contact Tracing and Notification: Navigating Six Points of Failure

**DOI:** 10.2196/49560

**Published:** 2023-12-04

**Authors:** Joanna Masel, James Ian Mackie Petrie, Jason Bay, Wolfgang Ebbers, Aalekh Sharan, Scott Michael Leibrand, Andreas Gebhard, Samuel Zimmerman

**Affiliations:** 1 Department of Ecology & Evolutionary Biology University of Arizona Tucson, AZ United States; 2 Department of Applied Mathematics University of Waterloo Waterloo, ON Canada; 3 Pandemic Sciences Institute Nuffield Department of Medicine University of Oxford Oxford United Kingdom; 4 Big Data Institute Nuffield Department of Medicine University of Oxford Oxford United Kingdom; 5 Saw Swee Hock School of Public Health National University of Singapore Singapore Singapore; 6 Erasmus School of Social and Behavioural Sciences Department of Public Administration and Sociology Erasmus University Rotterdam Rotterdam Netherlands; 7 Aarogya Setu Government of India New Delhi India; 8 Community Epidemiology in Action (coEpi) Seattle, WA United States; 9 Temporary Contact Number Protocol (TCN) Coalition New York, NY United States; 10 PathCheck Cambridge, MA United States

**Keywords:** COVID-19, SARS-CoV-2, pandemic preparedness, decentralized protocols, smartphone, mobile phone, contact tracing

## Abstract

Digital contact tracing and notification were initially hailed as promising strategies to combat SARS-CoV-2; however, in most jurisdictions, they did not live up to their promise. To avert a given transmission event, both parties must have adopted the technology, it must detect the contact, the primary case must be promptly diagnosed, notifications must be triggered, and the secondary case must change their behavior to avoid the focal tertiary transmission event. If we approximate these as independent events, achieving a 26% reduction in the effective reproduction number R_t_ would require an 80% success rate at each of these 6 points of failure. Here, we review the 6 failure rates experienced by a variety of digital contact tracing and contact notification schemes, including Singapore’s TraceTogether, India’s Aarogya Setu, and leading implementations of the Google Apple Exposure Notification system. This leads to a number of recommendations, for example, that the narrative be framed in terms of user autonomy rather than user privacy, and that tracing/notification apps be multifunctional and integrated with testing, manual contact tracing, and the gathering of critical scientific data.

## Introduction

Contact tracing is a time-tested tool to fight an emerging outbreak of infectious diseases such as COVID-19, caused by the virus SARS-CoV-2. If x% of infections are identified and y% of those in contact with a known case are traced in time and persuaded to stay home, then in a well-mixed population, the effective reproduction number R_t_ will decrease by a factor of x×y. This seems sufficient to reach the level R_t_<1 needed to quash an outbreak of many emerging pathogens (which have not yet evolved a basic reproduction number R_0_>>1) before the outbreak develops too far, especially in combination with modest social distancing. However, SARS-CoV-2 proved to be a particular challenge for contact tracing. With short incubation periods and presymptomatic transmission making it difficult to trace contacts in time, digital contact tracing held tremendous promise, especially when leveraging smartphones that were already in consumers’ hands. Here, we reflect on why digital alternatives to traditional contact tracing instead had limited impact on SARS-CoV-2 transmission. We do so with a focus on Exposure Notification (EN) and related protocols, in part by drawing on first-hand experiences from our various involvements with this technology, including material that has not previously been public as well as publicly available documents that are not indexed for literature searches. Our intent is to better inform those who might wish to prepare for and fight a new pandemic with a similar technological approach, allowing them to learn from what happened during the COVID-19 pandemic.

## A Brief Note on Terminology

Some digital protocols make it impossible to identify pairs of interacting individuals, even in cases where one transmitted disease to the other—in this case, we refer to *notification* instead of *tracing*. We refer to *proximity* versus *presence* notification and tracing based on whether exposure is assessed on the basis of proximity to an individual versus presence at a shared-air venue. We reserve the term EN for the specific protocol implemented by Apple and Google; this protocol is a form of proximity notification. EN is one of several protocols used to assess proximity based on signals sent and received between pairs of devices using low-energy Bluetooth. Other protocols may use ultra wideband or ultrasound for proximity detection, or detect presence from other information such as QR code scan histories, GPS coordinates, or logs of Wi-Fi access points. These technologies can also be used to warn of future infection risk [1], a use case that is beyond the scope of this study.

## Overview of the Six Failure Points

**Figure 1:**
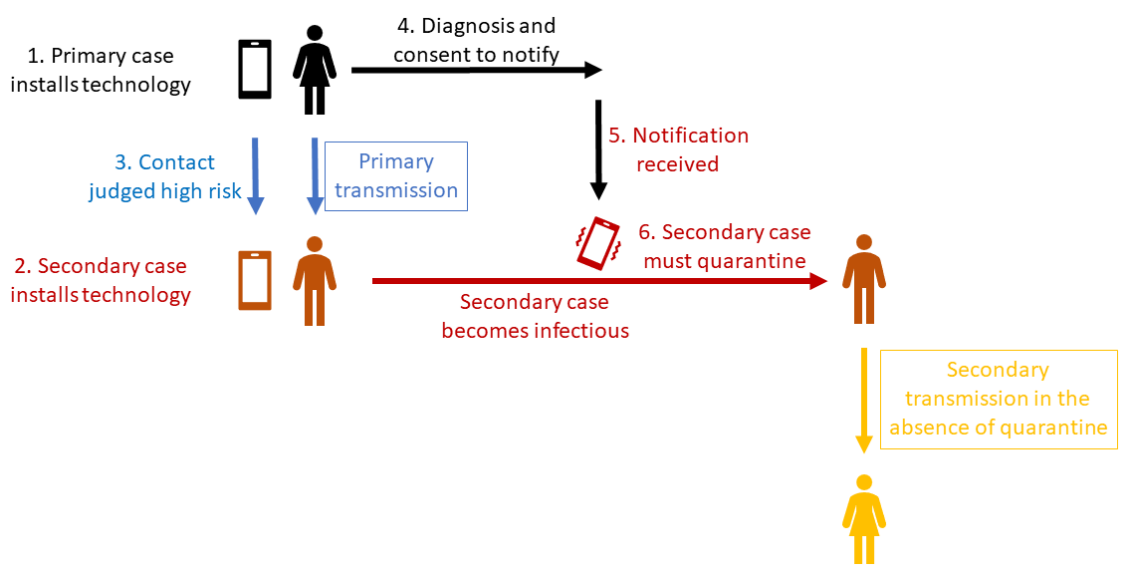
Chain of viral transmission from primary to secondary to tertiary case. Digital contact tracing and notification must succeed at all 6 critical points to avert transmission to the tertiary case.

To be effective in stemming a transmission, a notification must navigate six potential points of failure (Figure 1):

1. The primary case must have the technology in place at the time of transmission.
2. The secondary case must have the technology in place at the time of transmission.
3. The exposure that resulted in transmission must be judged to be high risk.
4. The primary case must obtain a positive diagnosis in a timely manner.
5. Notifications stemming from the primary case must be rapidly triggered following a positive diagnosis.
6. After receiving a notification, the secondary case must change their behavior in a manner that prevents onward transmission to tertiary cases.

If each of these steps were successful 80% of the time, and we approximate the 6 steps as independent events, then transmission (R_t_) would be reduced by 0.8^6^=26%. Although not transformational on its own, this would be a significant contribution to quashing an outbreak, and nonindependence will make this figure somewhat higher. However, if each step were successful a still-respectable 40% of the time, again assuming independent events, R_t_ is reduced by only 0.4%. Although this can make a valuable contribution to flattening a curve [2] or reducing the stringency of indiscriminate social distancing [3], it falls far short of containing a pandemic. Given this simple mathematical consideration, if the aim is containment such that life is relatively normal while waiting for a vaccine, then we clearly need to achieve low failure rates at all of the 6 failure points.

Next, we discuss each of the failure points. We provide some history of how they were handled, together with speculation about how they might have been handled better.

## Failure Points 1 and 2: App Adoption

We jointly discuss the first 2 points of failure because they are similar; both involve technology adoption among the population destined to become infected. Following media coverage of the model by Ferretti et al [4], more attention was paid to app adoption than to other points of failure, with an odd obsession with 60% adoption in the general population as a magic number [5]. Note that the adoption rate that matters is not that of the general population but that of the population likely to become infected. For SARS-CoV-2, this meant that adoption among essential workers was what mattered during stay-at-home mandates; when staying at home was common but optional, young adults constituted a disproportionate share of cases. However, app adoption among the general population can serve as a rough approximation.

The most straightforward way to achieve low failure rates at points #1 and #2, while still maintaining user autonomy, is to make the broadcasting and reception of appropriate signals opt-out. Opt-in consent of the primary case would still be required at failure point #5 and that of the secondary case at #6. Apple and Google were the only entities with the power to implement an opt-out protocol, with competitors such as Huawei having little market share. Despite a secure EN design that ensures that no information about Bluetooth signals sent or received leaves the phone without subsequent user consent, they chose to make Bluetooth broadcasting and receiving opt-in. We note that Apple’s “Find my...” service uses Bluetooth in a similar fashion and is at the time of writing opt-out rather than opt-in [6].

An even lower failure rate could have been achieved by a protocol proposed by one of us (JIMP) to use the Wi-Fi logs already collected by Android and by Apple’s iOS operating systems instead of an additional purpose-built Bluetooth layer. Had Google and Apple chosen to allow an app post hoc access to these logs or to build operating system functionality around them, this would have completely eliminated failure point #1 for individuals who regularly carry smartphones. Individuals would still need to have the technology switched on to bypass failure point #2 to receive a notification. Similar to Bluetooth solutions, those who do not regularly carry smartphones (mostly young children and older adults) would need to be provided with other devices that could record proximity or presence; in Singapore, small digital tokens resembling keychain fobs covered smartphone nonusers who would not otherwise have been able to participate.

To understand why Apple and Google did not make the technology opt-out or Wi-Fi based, it is important to consider the incentive structure from Apple’s and Google’s point of view. Cooperation requires time from their employees and has no apparent commercial upside (beyond the economic gains to the company should efforts succeed in containing a pandemic). From their perspective, the primary consideration is the effect of their actions on brand perception. The upside impact on their brand is gained primarily from doing *something* (eg, working together), but there is no proportionate impact from doing something more rather than less effective. The potential for downside impact is substantial and focused mostly on privacy concerns, exacerbated by any ceding of control over the technology and its marketing to public health authorities. They are thus incentivized to do *something*, to carefully manage perceptions of that something, and to avoid brand risk, but not to increase effectiveness against disease transmission. In future, if companies such as Apple and Google are to be gatekeepers of such technology, governments should devote urgent attention to aligning their incentive structures.

Achieving high opt-in adoption was difficult. Singapore’s non-EN solution succeeded, with opt-in adoption >90% even before the app became required for entry into public spaces [7]. In contrast, the highest EN adoption rates are of Germany’s Corona-Warn-App, used by approximately 34% of the population [8], and the National Health Service (NHS) COVID-19 app, with 17% to 25% of the population of England and Wales having activated Bluetooth exchanges in 2020 and 2021 [9]. Lower rates were reported elsewhere: 8% in Canada, 13% in New Zealand, 10% to 16% in the Netherlands, and 19% in Switzerland [10].

A positive caveat to this fairly bleak assessment of uptake is that if one individual has adopted the app, it is more likely that other members of their social network have also adopted it. In other words, failures at points 1 and 2 are correlated, making the probability of overall failure lower than it would be if each failure were an independent event [11]. Both for this reason and as part of good marketing practices [12], it can make sense to look at adoption rates within smaller communities. Adoption by 46% of the cases was achieved in a campus setting with an intensive marketing push [13] and adoption by >33% of the population was achieved in an island setting [14].

Key to the relatively high adoption of the NHS COVID-19 app and the Corona-Warn-App were their additional functionalities. When entering a venue, scanning a QR code with the NHS COVID-19 app was offered as an alternative to writing down contact details. Although subsequent presence tracing on the basis of either form of information was conducted relatively rarely (refer to *Failure Point 5:* *Triggering Notifications* section), the requirement to check in to a venue did prompt app installation [15], especially in the subset of the population most likely to become infected through contact with strangers. The NHS COVID-19 app was also useful for ordering tests, and both it and the Corona-Warn-App offered a rapid and secure system for receiving test results. Later, the integration of European Union digital COVID-19 certificates (for vaccination, recent negative test result, or recovery from infection) triggered another wave of app adoption. Near-universal adoption in Singapore was achieved only after the proximity tracing app became the only means for compulsory venue check-in [16]. Similarly, adoption rates in India skyrocketed after the government made the app mandatory for smartphone owners to move freely in public areas. We believe that to achieve high adoption, future pandemic apps need to seamlessly integrate services that are useful and convenient for users.

In late 2020, Apple and Google launched the “Exposure Notification Express” (ENX) system. In Androids, this is a simple app that public health authorities can autogenerate from a small set of choices. In Apple’s iOS, this is part of the Settings. Apple claimed that placing the scheme in the Settings created less friction for opt-in than was present for an app, and hence, ENX should be preferred to a custom app. However, ENX never reached use levels comparable with those of more successful European EN apps. This is despite the fact that the phones of residents of adopting US states were pinged to promote activation and installation. Such pings were used to promote ENX but not custom EN apps. When this situation was pointed out, Google immediately offered the same adoption promotion pings for custom apps as for ENX, whereas Apple declined to do so.

A number of players, including but not limited to Apple, argued that the key to persuading people to adopt lay in ensuring the privacy of the system. Fortunately, decentralized protocol designs offer powerful solutions to the privacy problem. Centralized approaches send information about who went where with whom to a central database. This breach of privacy, although clearly facilitating contact tracing, also poses risks of abuse as part of a mass surveillance state. Under a decentralized protocol, information about a user is stored on that user’s device and, to some degree, the devices with which it was in contact. Users can be given autonomy over the use of data on their own devices so that, for example, the data cannot be accessed without consent and can be deleted at any time. Far from threatening privacy, apps that use decentralized protocols are among the safest apps on users’ smartphones.

Interestingly, privacy-invasive schemes, such as QR code check-in presence tracing in Australia, were well accepted and widely used (self-reports of 61.9% always checking in either digitally or on paper, 26.3% mostly, 4.7% sometimes, 3.3% occasionally, and 3.8% never [17]). This achieved acceptably low failure rates far superior to any EN implementation, even with enforcement left to the venue and peer pressure. Indeed, supported by celebrity-driven public relations pushes, 40% had downloaded or were willing to download even the ineffective but convenient Luca QR code check-in app in Germany [18,19]. Perhaps, these much higher adoption rates for presence tracing than for proximity notification are because what was recorded was considered public information that a user was in a public space, rather than also capturing who had interacted with whom even in private. A simpler explanation is that the QR code check-in system is easy to understand as equivalent to writing down your name and number; decentralized privacy-preserving schemes, by being harder for the public to understand, run the paradoxical risk of decreasing rather than increasing trust and hence adoption.

Trust in government predicts adoption more strongly and consistently than privacy [15,20-23]. Although 20% to 25% of respondents to cross-sectional surveys cited privacy concerns as a reason for not installing a contact tracing app [23,24], a longitudinal study found no causal relationship (although it did for health concerns and social norming) [25], and a focus group found that health concerns were more important than privacy considerations [26].

High trust in the government in general [27] as well as in the government’s response to the COVID-19 pandemic [28] was critical to Singapore’s high adoption. When trust in governments is low, it might help if more trusted entities, such as primary care providers [29], were used to promote adoption. Singapore’s explicit policy to relax certain pandemic restrictions once adoption increased from 50% to 70% might also have helped drive adoption, together with the scheme’s high profile as a clear government priority.

Privacy is not the only aspect of trust salient to potential users; many also wanted to know that an app was effective before they downloaded it. Even if they decided to take a chance at first and install it, they wanted subsequent reassurance that it had proved effective to keep it active on their phones. Multifunctional apps, such as the NHS COVID-19 app and the Corona-Warn-App, can also provide reassurance via interaction that there is a point to maintaining the app on the user’s phone. Although it is clearly important to hire good marketing and public relations professionals to promote adoption, it is just as important that scientists and engineers do not make product decisions in isolation from their impact on marketing. This is because achieving high adoption is part of the science of making these apps effective.

Distinct from the scientific study of app effectiveness in stemming transmission [30] is the study of viral transmission itself to learn its incubation period, infectious period, and mode of transmission [31]. An obstacle to such a study was that some *privacy-first* rhetoric rejected making the study of SARS-CoV-2 transmission an aim of the scheme, even if the science could be done in a manner that preserved privacy.

The only reliable way to learn about transmission to and from humans is to observe it, either in human challenge trials (directly observing incubation periods and also the infectious period if the study includes transmission to cohoused animals) or in natural circumstances (ie, via contact tracing). Given ethical concerns about the former, an unacknowledged corollary of rejecting the use of digital schemes to study transmission is that studies must be performed via manual contact tracing (ie, in a more privacy-invasive way) or not at all. Unfortunately, even the more basic functions of manual contact tracing were quickly overwhelmed, leaving little capacity for the more intensive investigations required to study transmission dynamics. Not using digital approaches to fill this void was a lost opportunity. This left early manual contact tracing studies as the only source for basic transmission parameters, even after the incubation time and infectious period of SARS-CoV-2 were suspected to have shifted because of viral evolution and immunity.

Even an app whose failure rates across the 6 points were too high to substantially reduce transmission could have been enough to generate valuable information about evolving incubation periods and infectious periods in close to real time [31]. Apps can also help with epidemiological surveillance; for example, the Indian government combined location (with a random error added for privacy; EN apps were banned from accessing location) and symptom information to identify emerging hot spots before seeing spikes in test positivity. Singapore used the number of detected contacts per case to inform changes in social distancing policies. Support for the scheme, both from the public and from public health authorities, might have been shored up by evidence that it was at least doing good for science or disease surveillance, and this might have created a virtuous circle of adoption.

A prevailing narrative described the technological choices in terms of a trade-off between privacy and effectiveness, in which any collection of more data can increase perceived privacy risk and hence reduce public acceptance [30]. We believe that this framing is harmful, especially when it comes to promoting app adoption; for example, the public might conclude that because the technology is private, it cannot be effective. Regarding substantive risks beyond those of perception, with flexibility and creativity, effectiveness can be achieved by a decentralized, privacy-preserving design that does not risk expanding mass surveillance. With sufficient ingenuity, there may be no need for compromise at all, which makes the privacy versus effectiveness trade-off narrative misleading. *Privacy first* should therefore not be used as grounds to support the status quo to the point of refusing to engage in ongoing dialog regarding how to safely proceed with iterative improvements to effectiveness, its measurement, and the gathering of broadly valuable scientific or epidemiological surveillance data. Given that harms from technology are not limited to privacy concerns [32], a privacy-preserving design does not relieve governments of their duty to monitor effectiveness [33].

Furthermore, privacy is merely a component and a means to the more important end goals of autonomy and protection from abuse. The latter need not be a serious issue because a well-designed, decentralized solution can offer both effectiveness and protection from abuse. However, trade-offs between effectiveness and autonomy are real, that is, coercion can increase effectiveness, whereas autonomy can weaken it. The option to maintain privacy while extracting benefit from the system is an important but not the only aspect of autonomy.

Although the privacy approach of EN provides excellent protection from abuse, it actually limits autonomy by denying users the right to share their data with public health authorities if they wish to. This became clear when one of us (JIMP) proposed a small change to the EN protocol that could convert it from proximity notification to proximity tracing, without significantly raising the danger of misuse. As described in the *Failure Point 3: Detecting and Evaluating Exposure* section, this modified protocol would allow certain users to share more data with public health authorities. This would apply to users who gave explicit consent to sharing after both receiving a notification and testing positive. By helping manual contact tracers do their job of pairing cases and identifying superspreading events, this change would presumably have generated more buy-in from public health authorities (discussed in the *Failure Point 5: Triggering Notifications* section). More buy-in from the local public health authorities responsible for manual contact tracing could have set up a virtuous circle by which they, in turn, promote adoption in the community. This small modification was rejected by Apple as being a threat to privacy, despite the fact that any privacy loss is triggered by users exercising autonomy over the use of their data. Contributing to this dynamic is the fact that privacy is part of Apple’s brand, aligned with the broader deployment of antitracking as a business strategy [34-36]. A preference for privacy over autonomy, by disallowing the opt-in sharing of more information with public health authorities, has also been expressed by some academia-based protocol designers [37].

The slogan *privacy first* is an odd one. We believe that autonomy and protection from abuse are better-framed goals than privacy. Furthermore, the best way to put privacy first is self-evidently to have no digital system at all, raising the possibility that privacy first schemes were designed primarily to displace more invasive options, with stemming transmission almost an afterthought. For each proposed scheme, one should assess the nature and scope of any vulnerabilities versus the anticipated impact on disease transmission.

## Failure Point 3: Detecting and Evaluating Exposure

Point of failure #3 concerns the need to record an interaction at which viral transmission took place, but viewing it this way is not sufficient. Total success could trivially be achieved by a general stay-at-home order, which is equivalent to telling everyone that they are potentially exposed. This illustrates the importance of avoiding false positives, that is, alerts received by people who are not infected. The primary purpose of proximity or presence notification is best seen as identifying and alerting people at a sufficiently high risk of infecting other people such that they should change their behavior in response to that information. This benefit applies only to secondary cases who would not otherwise know of their exposure in a timely manner, that is, it excludes household contacts and other social networks that perform rapid do-it-yourself tracing. A major advantage of digital schemes is their complementary ability to notify strangers.

One aspect of this is to detect any contact at all. With core (ie, regular) Bluetooth, iPhone-to-iPhone communication does not work in the background. Apple’s cooperation in setting up EN was needed to overcome this serious obstacle. Apple could have chosen to enable iPhone-to-iPhone communication in the background in the same way that they already allowed background communication between iPhones and Androids, which would have allowed app developers to implement their own protocols. Indeed, India launched the Aarogya Setu app using core Bluetooth rather than EN, given that iPhones are rare in India compared with Androids. Singapore stuck to its own BlueTrace protocol (based on core Bluetooth), given the sacrifice of epidemiological utility that switching to EN would have implied [38], eventually leveraging Android-iPhone background communication into a gossip protocol that effectively allowed iPhones to see each other in the background whenever an app-using Android was also present [39]. Other non-EN jurisdictions such as France pivoted away from proximity notification and toward presence tracing. In Australia, state-specific centralized presence tracing apps were the dominant response, whereas a federal app based on TraceTogether, despite a respectable early download rate, languished once it became apparent that it failed at other steps.

The lack of interoperability among jurisdictions can also interfere with contact detection. For example, if each person is far from home for 5% of their interactions (eg, being away for 18 d/y), the corresponding contact detection failure rate will be approximately 10%. Thus, when viewed quantitatively, the considerable attention devoted to this issue seems disproportionate relative to other sources of failure. As an alternative to immediate global standardization, metropolitan or other highly interconnected areas spanning multiple jurisdictions (eg, Washington, District of Columbia combined with adjacent counties in other US states or the Navajo Nation spanning multiple US states) could choose to roll out a joint product. Multiple apps need not interfere with each other, making this an option for some commuters. A common server was set up to enable communication among the EN implementations of different US states and another common server to communicate among implementations of different European nations [40], but despite the attention to this issue, global interoperability was never achieved. Perhaps the best model for a new pandemic is to allow divergence in the early stages as part of a process of innovation at the cost of interoperability, to learn from the different experiments performed by different jurisdictions, and then to use the desire for interoperability as the catalyst for switching from lower-performing systems to higher-performing systems through app updates. An advantage of postponing interoperability is to ensure that premature standardization does not suppress innovation.

Beyond detecting exposure at all, doing a better job in assessing the infection risk posed by a given exposure can be seen as an ethical imperative, given the harms caused by unnecessary quarantine [32]. Better risk analysis means more precise resolution as to which individuals pose how much statistical risk to others on a given day and using a risk threshold to trigger notification. In other words, good risk analysis means improving the receiver operating characteristic (ROC) curve with respect to infection and then choosing a socially optimal point along the ROC curve.

Under reasonable assumptions, the socially optimal approach is to alert those who are significantly more likely to infect others than is the average member of the population not already in quarantine or isolation [3]; for the NHS COVID-19 app, notified individuals were 2- to 20-fold more likely to subsequently report a positive test [41]. Under the assumption that some form of regulation maintains a geometric mean of approximately R_t_=1 while waiting for a vaccine, the total value of both manual contact tracing and digital notification schemes is highest at low case prevalence [3] when fewer individuals need to quarantine to achieve the same population benefit. Digital contact tracing also becomes more important when social restrictions are few, and interactions among strangers are thus more common. Unfortunately, in many jurisdictions, attention to all pandemic measures tended to rise and fall with case counts rather than fluctuating between a focus on contact tracing during lulls and on population-wide measures when case counts were high. This interfered with effective planned implementation.

One obstacle to good risk analysis was public health guidance that ignored evidence for airborne transmission in favor of the droplet theory that physical distancing of 2 m (or 1.5 m or 6 feet) was effective protection. As a result, significant technological research focused on calibrating EN Bluetooth settings to a threshold of 2 m or similar [42-45]. A number of unpublished analyses suggest close to superimposable ROC curves, regardless of Bluetooth settings. Superior distance assessment via Bluetooth could likely have been achieved using all 3 Bluetooth channels instead of 1 or by using the median attenuation instead of the mean (decisions made within the EN application programming interface [API] and hence not available to app developers). Ultra wideband or ultrasound would assess the distance more precisely than Bluetooth. However, none of these tweaks change the fact that distance only moderately predicts infection risk, and proportionate effort was not invested in integration with predictors such as local carbon dioxide levels. Nor were users provided with information such as the time or location of exposure, although this information was present on their phone. This prevented users from integrating information about masking and ventilation into their personal assessment of the risk of infection. Not allowing this, even with the permission of the primary case, is another example of privacy being put before autonomy.

After coming up with *good enough* Bluetooth settings, a better approach would have been to deploy an app that collected data both on the parameters of exposure and on whether it was followed by infection. These data could have informed the risk settings better than any experiment on Bluetooth-distance relationships. We note that a protocol might flag an individual as high risk on the basis of an exposure other than the one that infected them; real-world calibration will include such cases, potentially increasing impact beyond that of causal connections.

An ongoing process of data collection and risk calibration would have taught us much about transmission, for example, any changes to the timing of infectiousness as new strains appeared and as individuals acquired prior immunity. This is important because exposure dose, and hence infection risk, depends not only on the physical characteristics and duration of an encounter but also on how infectious the primary case is at that moment. The main data used to assess this were the date of symptom onset or, if asymptomatic, the date of the first positive test. The infectiousness window was initially estimated to run from approximately 2 days before to ≥5 days after symptom onset [46]. Unfortunately, a bug in the code of the original analysis concealed the earlier onset of infectiousness. Although this was rapidly identified and corrected [47,48], public health guidelines, describing the days for which the primary case should be considered infectious for contact tracing purposes, did not change accordingly. Nor did they modify the contact tracing window following the emergence of new SARS-CoV-2 variants, nor for reinfection or breakthrough cases. To re-estimate this window, they would need to repeat the same type of intensive manual contact tracing studies; this did not occur. Other information about infectiousness could, in principle, come from the Ct count (which was not reported in standard laboratory SARS-CoV-2 polymerase chain reaction testing protocols) or connection to a superspreading event, but neither was used in practice.

EN version 1 allowed the app that is installed on the smartphone of the primary case to assign 1 of 8 levels of infectiousness to each day during which others might have been exposed. This integer is the only metadata regarding the primary case that is available to the exposed contact. EN version 2 enabled some improvements to risk analysis by lifting the previous 30-minute cap on exposure reported by the contact’s phone but reduced the number of levels of infectiousness to 2 (although Germany repurposed other bits of information to reclaim the use of 6 [49]). When asked to make the infectiousness metadata easier to use for both risk analysis and scientific study, specifically to propagate the timing of exposure relative to symptom onset or test date, from the server to the exposed contact, Apple replied that they could not do so because of a vulnerability whose details had been worked out by Google but which they could not remember.

Beyond EN, some jurisdictions acknowledged airborne transmission to the point of performing not just proximity tracing but also presence tracing. In corresponding presence tracing schemes, people going to venues such as restaurants would either write down their name and contact details or use an app to check in by scanning a QR code. For Singapore’s SafeEntry, they could also use a Bluetooth-based sensor (to detect a TraceTogether app or token) or scan a barcode. With a low-risk threshold, for example, a jurisdiction with a zero COVID-19 policy, all individuals who were present at the same venue as an infectious individual could be alerted.

A better strategy for a higher risk threshold is to focus on *cluster busting*, that is, prioritizing those venues at which at least 1 transmission event is already known to have occurred. These generally correspond to gatherings at which the primary case was highly infectious, ventilation was poor, and there were other risk factors such as speaking, singing, and exercising and lack of mask use. There are compelling arguments for the effectiveness of such *backward tracing* for pathogens such as SARS-CoV-2 [50-52]. This requires cases to be linked.

A decentralized protocol such as EN could have contributed to cluster busting, had the app of a user who was first exposed then tested positive been allowed to upload the cryptographic key responsible for triggering the exposure. EN chose to keep this key sequestered within the operating system, with no option to notify the server. If a key associated with a transmission event were uploaded, the infectiousness score of that key could then be significantly upgraded on the server, triggering follow-up notifications with stronger wording as well as additional notifications following briefer exposures to the possible superspreading event. If users were additionally given the option of sharing their identity with public health authorities, paired with the cryptographic key that served to link them, then both members of the pair could be prioritized for interviews and the location of the superspreading event identified to assist manual contact tracers.

## Failure Point 4: Test Access

No form of contact tracing, digital or manual, will work unless we are able to identify who is likely infected and, thus, whose contacts should be notified and traced. Although some countries such as South Korea did better, in many countries, SARS-CoV-2 tests were slow to roll out, testing capacity was quickly overwhelmed, test shortages persisted for a surprisingly long time, and not all symptomatic individuals sought testing [53-55]. It is obviously critical to improve pandemic preparedness in this regard, independent of its relationship with digital notification or tracing.

Improving the allocation of scarce testing resources by identifying which individuals were most likely to test positive was a primary use case for India’s Aarogya Setu, with the highest-risk individuals having positive predictive values >40%. To achieve this, Aarogya Setu used a sophisticated risk algorithm to trace not just direct contacts but also contacts of contacts and so on (multiple-degree tracing) in a risk-consistent manner, faster than testing could keep up [56]. In EN, functionality for contacts of contacts was limited, relied on self-reporting, and was never used. Another important factor for allocating scarce testing resources is utility for onward contact tracing and notification, for example, by abandoning samples more than a certain number of days past symptom onset to rapidly turn around tests whose results will be more actionable.

For some pandemics, there may be one or more hallmark symptoms (such as loss of taste and smell) that are sufficiently distinctive to warrant presumptive diagnosis. In other cases, the symptoms might be suggestive but less definitive. Strategies for triggering notifications given contact with an unverified case, in the absence of test results, overlap with the next point of failure, namely, how to manage the verification of positive test results.

The CoEpi app [57] attempted to launch on the basis of symptoms alone using core Bluetooth (it was designed before EN). It was rejected by the Apple Store on the basis of a lack of public health authority involvement, with the suggestion to seek public health authority support from a “city or county.” Its resubmission with explicit support and endorsement from the public health department of a county in Washington state (from a different nonprofit account because Apple suspended the primary developer’s account without explanation) was also rejected. Apple representatives told developers that unwritten and nonpublic rules stood in the way and that it was the desire of Apple to not have “competing” approaches.

When test turnarounds take several days, but each day is critical, it might be helpful to issue *preliminary* notifications on the basis of exposure to an exposed, symptomatic individual awaiting test results to be converted later to a confirmed exposure. The same principle can be expanded deeper into a social network. We note that recursive protocols can incidentally achieve some of the functionality of backward contact tracing, showing up as multiple social network paths via all attendees to the index case.

## Failure Point 5: Triggering Notifications

Following a positive diagnosis for SARS-CoV-2 in a primary case, speed is essential to notify secondary cases before they transmit onward to others. The simplest option is to allow any app user to self-attest that they are infected. An issue with this is the potential for abuse. For example, before an election, a coordinated set of individuals could intentionally socialize widely in a setting that tends to vote in a particular way, for example, a college campus, and then falsely report positive diagnoses timed to trigger quarantine on the day of the election. This was a specific concern for the US November 2020 elections. Disruption of essential workers at core infrastructure might also be achieved through targeted attack.

In this light, and to ensure trust in the system, all EN jurisdictions launched with systems that ensured that only verified positive test results could be used to trigger notifications. Many jurisdictions initially relied on manual contact tracers to issue a time-sensitive secure code over the phone to individuals who tested positive. This led to extremely high failure rates, given that case investigation was overwhelmed. EN imposed extra work on case investigators in issuing verification codes but provided nothing back to them in return, for example, by helping link cases into transmission chains, as could be done by the protocol tweak described earlier. Even when the code was successfully delivered and entered into an app, it tended to occur with a significant delay. This defeated much of the purpose of a digital scheme, whose motivation was to make contact tracing faster [58]. Primary care can similarly become overwhelmed, and in a controlled, nonoverwhelmed setting, voluntary adherence even once a code is provided was only 64% [14]. In contrast, Singapore’s solution was fully integrated with both testing and manual contact tracing, speeding up the latter from 4 days to <2 days [59] and switching to primarily automated alerts only after case counts increased during the Delta wave in late 2021.

Google and Apple insisted on having only 1 EN app per country or, in the case of the United States, 1 per state. However, test and trace programs are run by counties and tribal nations in the United States, by provinces in Canada, by cantons in Switzerland, and so on. This disconnect did not encourage buy-in by the local public health authorities charged with distributing secure codes; app design choices were made by a different level of government than the level responsible for implementing the test and trace policies.

Presumably, Google and Apple preferred to limit the number of relationships they needed to maintain and of EN apps whose code they needed to review. However, this could also have been solved via a flexible global app (or an ecosystem of several at least partially interoperable apps); Google and Apple would only need to deal with 1 or several app developers, who in turn would deal with the customizations requested by the various jurisdictions. Such an ecosystem began to spontaneously appear through players such as NearForm, PathCheck, and WeHealth. A bottleneck in many US states was the slow process of government procurement to pay such players. One reason many US states opted for ENX was that because no payment was required, it bypassed delays in the procurement process, despite the fact that running ENX and associated verification code distribution still generally required a state public health authority to internally dedicate full-time staff. With the advent of ENX, Google and Apple ended up needing to maintain more relationships than they would have with private-sector middlemen.

Given the difficulties in providing verification codes by phone, most US implementations, beginning with the state of Colorado, shifted to a system based on SMS text messages. Positive test results reported to the state were collated, and cases were then, in batches, each sent an SMS text message with a deep link that acted as a verification code. Vague language and a few decoy SMS text messages were used to comply with the Health Insurance Portability and Accountability Act. There was still some posttesting delay associated with reporting and collating, some SMS text messages were caught by spam filters, some were lost to the SMS delivery network during peak use periods, and the system was confusing for recipients who had never heard of EN. However, it was faster and had a higher success rate than having a manual contact tracer issue a code over the phone. The ratio of #claims/#cases (measuring a combination of failure points 1 and 5) went up, for example, from 1.8% to 9.6% in the state of Washington (with the caveat that both are upper bounds on success because the total neglects delays in receiving codes and includes codes claimed by individuals who installed EN only after receiving the deep link) [60].

An alternative way to issue verification codes was to integrate them with testing, that is, with the health care system rather than the public health system. The best solution is to make the app into a test result delivery system, that is, to link test samples to QR codes, with the app knowing the ID of its user’s test and checking a server for matches. This shortens the time from a sample testing positive in the laboratory to notifications being triggered. It still requires the user to check their notifications for a positive test result and to consent to have their contacts notified. In Singapore, the median time from SMS text message notification of test results to consent to upload data was <30 minutes, with a consent rate of 70% to 80%. EN was eventually modified to allow an even better preauthorization workflow, whereby consent could be given at the time of testing, and notifications were sent as soon as the app became aware of the positive test result, with no further user input required. This system was used in Germany. Germany also offered free supervised antigen tests; the unvaccinated required a recently verified negative result to permit access to some venues, and positive results could be verified by the same system for use in EN.

Integration with testing was easier for the NHS COVID-19 app than for many others, given an already centralized system. However, it was also possible elsewhere; Germany integrated its app with >10,000 different test providers. The speed and convenience of obtaining a polymerase chain reaction test result back through the app helped prompt download, although some users opted only to use this feature and not to trigger notifications. It is important to investigate the cause of this refusal, for example, did it stem from distrust regarding anonymity, were people trying to avoid pushing friends and family into uncompensated quarantine, or was it simply an instinctive “no” under time pressure in a stressful situation, for which a different workflow might have elicited a different decision?

Two EN pilots at US universities explored integration with campus testing programs, which provided rapid test turnaround before adequate testing became available to the general public. The University of Alabama linked test results to phone numbers and then allowed individuals to use their phone number as their verification code through the use of a 1-way hash. The University of Arizona provided verification codes as part of the web-based portal from which the test results were distributed, with 25% of cases claiming a verification code [13]. As part of an (abortive) expansion to the state at large, API integrations were created not only with the state’s largest test providers (LabCorp, SonoraQuest, and others) but also with Doximity, an app believed to be used by 70% of the state’s physicians. Surveys show higher trust in health care providers than in public health authorities [29,61], making physicians’ involvement potentially useful.

In retrospect, all these schemes were too complex to work reliably from the outset, at least in most jurisdictions. Better might have been to allow self-attestation of positive test results or even self-attestation on the basis of symptoms alone at times during which tests are in short supply (refer to the *Failure Point 5: Triggering Notifications* section). To avoid the potential for malicious use, unverified reports could trigger notifications with different messaging: not requiring quarantine, merely warning the recipient, and including the fact that the person who exposed them had self-attested to their infected or symptomatic status. Once postexposure quarantine was relaxed and home testing became the norm, many EN jurisdictions switched to self-attestation.

All the same difficulties arise with presence tracing. Many QR code check-in schemes required manual contact tracers to identify locations of interest to trigger app notifications. Similar to other forms of manual contact tracing, including pen-and-paper check-in at venues, this process was often overwhelmed. Although fully automated systems would have been faster and more reliable, they were not permitted for apps that also ran EN. New Zealand implemented this functionality early on, that is, users who tested positive were able to upload the set of QR codes they had scanned to public health authorities. However, when the NHS COVID-19 app attempted to follow suit, its app update was rejected [62], and New Zealand was also forced to make changes. Google and Apple allowed EN app users to store a local copy of their check-in history on their phone and to read it over the phone to manual contact tracers, but they did not allow an opt-in upload button, which is again a rejection of user autonomy on the grounds of privacy. They did permit an alternative automated QR code check-in system in the German EN app that was designed to never be linked to identities; however, because this system did not satisfy laws in the many German states requiring the collection of identity information, this limited its adoption.

Finally, many systems assumed that once an individual tested positive, they would enter isolation such that only past contacts and not future contacts would need to be notified. This turned out to be overly optimistic. We recommend that future systems anticipate nonadherence, with daily prompts of “Did you succeed in isolating today?” leading to the option to upload new data to anonymously notify that day’s contacts, in addition to contacts from the initial upload.

## Failure Point 6: Behavior Change

To stem transmission, notification needs to result in behavioral change by secondary cases. Rates of quarantine can be low, estimated at 28% for asymptomatic individuals in Norway adhering for at least 1 day [63], and 11% in the United Kingdom for quarantine adherence on all recommended days [64] (albeit much higher in the app-using subset [65]). Quarantine adherence was 40% among the app-using subpopulation in the Netherlands [61]. Adherence increases with trust in the government’s response to a pandemic [66].

Short of outright coercion, quarantine adherence might be improved by paying people, whether paid directly by the government or via employer mandates. Adherence might also be higher if, out of respect for their time, rigorous risk analysis (including the use of negative tests) were used to reduce quarantine duration to a minimum, with a risk threshold set on a rational basis. Some European countries gave quarantine pay to individuals traced by contact tracers but not those notified by EN. It is important to make such choices rationally, that is, to estimate the positive predictive value of an EN with given characteristics and to treat individuals with the same risk of infecting others similarly, regardless of the mode of risk detection.

There can still be substantial utility to issuing low-exposure warnings to individuals whose positive predictive value does not warrant quarantine. Short of full quarantine, more modest behavior changes also help stem transmission, for example, testing and isolating if positive [67], being more alert for symptoms and isolating if they appear, mask wearing, or avoidance of large groups and individuals who are vulnerable to severe disease. However, adherence to even these more modest requests is also far from universal, for example, in the state of Washington, only 40% of the subset of EN recipients who responded to a survey intended to get tested and 67% intended to watch for symptoms; rates were 58% and 84% on the smaller subset that also replied to a follow-up survey about actual behaviors [68]. With Paxlovid or other antivirals most effective when taken early, we note that even if notification fails to stem transmission, it can be of direct benefit to an individual who makes no changes other than monitoring for symptoms, then testing and treating if positive. Adoption rates (points of failure 1 and 2) might be higher if this direct benefit were stressed rather than relying on altruistic motives.

Even without complexities such as recommending quarantine for some but not all exposures, effective communication of *next steps* at a low reading grade level is surely important for adherence. In contrast, some EN implementations launched with quarantine recommendations that did not specify an end date, leaving users to assume that it began with the date of notification receipt rather than the unknown date of exposure. Other recommendations gave the date of exposure and left the user to perform the calculation of the quarantine end date.

Although the purpose of this piece is to present lessons learned, the better to inform responses to the next airborne pandemic, we note that the SARS-CoV-2 pandemic remains unpredictable in its evolution and that EN has not yet been sunsetted in all jurisdictions. In that light, we note that the end of quarantine does not mean the end of EN. If notification caused individuals to mask, avoid those most vulnerable to severe disease, or test then isolate if positive, then the technology would still be doing something useful, should sufficiently low rates of failure at stages 1 to 5 be achieved.

## Governance for Effectiveness

To contain a pandemic, that is, to achieve R_t_<1 through digital tracing and notification, an implementation must keep all 6 failure rates low, for example, <20%. This will require good governance, for which it can be instructive to learn from the best implementations thus far. The NHS and Germany had among the best EN implementations, even though their failure rates remained far higher than this benchmark. Singapore’s non-EN implementation was more successful, with a failure rate of <5% for failure points 1 and 2 and acceptably low failure rates for 4 and 6. We hope that more scientific data might eventually emerge from it.

It is notable that both the German and NHS COVID-19 app projects engaged not only software developers, privacy experts, and applied public health practitioners but also well-respected academic epidemiologists with significant track records of directly related research and that these epidemiologists had substantial (but not sole) influence on decision-making. They might be in the best position to balance ambition of scope (to achieve R_t_<1 not “we did something”) with realistic expectations about how things will play out on the ground. This follows a more general pattern during the COVID-19 pandemic in which applied public health institutions such as the Centers for Disease Control and Prevention performed poorly, but many universities and research institutions such as the Robert Koch Institut performed relatively well, as did biosecurity-run initiatives such as Operation Warp Speed, and the pharmaceutical industry when given a good incentive structure.

The fact that iPhone-to-iPhone low-energy Bluetooth communication did not initially run in the background meant that some response from Apple was needed for effective Bluetooth-based proximity tracing and notification using iPhones. Once that response took the form of EN, countries that did not capitulate to Apple’s extensive conditions for access often ended up focusing on presence tracing and notification systems instead (eg, France and Australia), unless they used a gossip protocol (Singapore) or issued alternative hardware (Singapore) or had few iPhones in their country (India). Apple’s ability to exert power over EN protocol design and over API access substantially restricted the scope for innovation among individual apps. Whatever the best form of governance is, few would argue that it is best done behind the closed doors of a technology company. Given that Apple exerted its power to dictate terms and limit innovation, it is a tragedy that Apple did not use its power to enact an opt-out system. For example, the NHS COVID-19 app is estimated to have saved 10,000 lives in its first year [41], and it is estimated that for every 1% increase in uptake, it could have reduced cases by a further 1% to 2% [2].

Future success will require the measurement of failure rates at all 6 points and rapid on-the-fly adaptations to improve them. Ideally, we would invest now in these technologies, perhaps within island nations or other close-knit communities as test cases, to iteratively improve systems as part of pandemic preparedness while at the same time attempting to reduce SARS-CoV-2 and potentially influenza transmission in the short term. An iteratively improved technology is more likely to be successfully deployed should a new pathogen begin transmitting between humans, one that combines Severe Acute Respiratory Syndrome–1 or influenza A virus subtype H5N1 mortality with SARS-CoV-2 presymptomatic transmission. In the absence of current investment, we hope that this document will help kickstart the design of effective strategies at such a time.

## Abbreviations

API: application programming interface
EN: Exposure Notification
ENX: Exposure Notification Express
NHS: National Health Service
ROC: receiver operating characteristic
